# Structure-Activity Relationships of the Bioactive Thiazinoquinone Marine Natural Products Thiaplidiaquinones A and B

**DOI:** 10.3390/md13085102

**Published:** 2015-08-10

**Authors:** Jacquie L. Harper, Iman M. Khalil, Lisa Shaw, Marie-Lise Bourguet-Kondracki, Joëlle Dubois, Alexis Valentin, David Barker, Brent R. Copp

**Affiliations:** 1 Malaghan Institute of Medical Research, PO Box 7060 Wellington South, New Zealand; E-Mails: jharper@malaghan.org.nz (J.L.H); l.shaw@centenary.org.au (L.S.); 2 School of Chemical Sciences, University of Auckland, Private Bag 92019, 1142 Auckland, New Zealand; E-Mails: ikha019@aucklanduni.ac.nz (I.M.K.); d.barker@auckland.ac.nz (D.B.); 3 Laboratoire Molécules de Communication et Adaptation des Micro-organismes, UMR 7245 CNRS, Muséum National d’Histoire Naturelle, 57 rue Cuvier (C.P. 54), 75005 Paris, France; E-Mail: bourguet@mnhn.fr; 4 Institut de Chimie des Substances Naturelles, CNRS UPR 2301, Centre de Recherche de Gif, Avenue de la Terrasse, 91198 Gif sur Yvette Cedex, France; E-Mail: joelle.dubois@icsn.cnrs-gif.fr; 5 Université Paul Sabatier, PHARMA-DEV, UMR 152 IRD-UPS, Université de Toulouse, 118 Route de Narbonne, F-31062 Toulouse cedex 9, France; E-Mail: alexis.valentin@univ-tlse3.fr

**Keywords:** thiaplidiaquinone, *Aplidium*, ascidian, thiazinoquinone, apoptosis, Jurkat, cytotoxicity, malaria, farnesyltransferase

## Abstract

In an effort to more accurately define the mechanism of cell death and to establish structure-activity relationship requirements for the marine meroterpenoid alkaloids thiaplidiaquinones A and B, we have evaluated not only the natural products but also dioxothiazine regioisomers and two precursor quinones in a range of bioassays. While the natural products were found to be weak inducers of ROS in Jurkat cells, the dioxothiazine regioisomer of thiaplidiaquinone A and a synthetic precursor to thiaplidiaquinone B were found to be moderately potent inducers. Intriguingly, and in contrast to previous reports, the mechanism of Jurkat cell death (necrosis *vs.* apoptosis) was found to be dependent upon the positioning of one of the geranyl sidechains in the compounds with thiaplidiaquinone A and its dioxothiazine regioisomer causing death dominantly by necrosis, while thiaplidiaquinone B and its dioxothiazine isomer caused cell death *via* apoptosis. The dioxothiazine regioisomer of thiaplidiaquinone A exhibited more potent *in vitro* antiproliferative activity against human tumor cells, with NCI sub-panel selectivity towards melanoma cell lines. The non-natural dioxothiazine regioisomers were also more active in antiplasmodial and anti-farnesyltransferase assays than their natural product counterparts. The results highlight the important role that natural product total synthesis can play in not only helping understand the structural basis of biological activity of natural products, but also the discovery of new bioactive scaffolds.

## 1. Introduction

**Figure 1:**
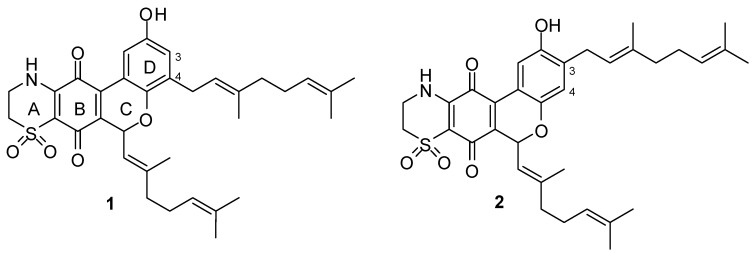
Structures of natural products thiaplidiaquinone A (**1**) and thiaplidiaquinone B (**2**).

The structures of marine natural products continue to provide not only targets for total synthesis but also new templates for biological evaluation. Ascidians of the genus *Aplidium* are a notable source of structurally complex natural products, including meroterpenoids, many of which exhibit potentially useful biological properties [1]. In addition to our own discoveries of rossinone B (potent cytotoxin) from an Antarctic *Aplidium* sp. [2] and scabellone B (antimalarial) from a New Zealand collection of *Aplidium scabellum* [3], Fattorusso’s group reported the structures of thiaplidiaquinones A (**1**) and B (**2**), two thiazine-meroterpenoids, from Mediterranean specimens of *Aplidium conicum* (Figure 1) [4]. Both **1** and **2** exhibited cytotoxicity towards the human leukemia T cell line Jurkat with IC_50_ ~ 3 μM with propidium iodide staining and flow cytometry data indicating that the natural products increased the frequency of subdiploid (apoptotic) cells caused by a rapid overproduction of intracellular reactive oxygen species (ROS) which in-turn led to a collapse of the mitochondrial transmembrane potential.

The interesting structures of thiaplidiaquinones A and B and their associated biological activities piqued the interest of ourselves and others, leading to reported biomimetic syntheses of **1** and **2** [5,6]. In addition to natural products **1** and **2**, our synthesis also led to the corresponding dioxothiazine isomers **3** and **4**. To investigate elements of the structural basis of ROS generation and Jurkat cell line cytotoxicity reported for the isolated natural products **1** and **2** [4], we have evaluated **1**–**6** in a similar set of assays and have also included more comprehensive evaluation for antitumor activity at the National Cancer Institute (USA) as well as for antimalarial and anti-farnesyltransferase activities.

## 2. Results and Discussion

### 2.1. Chemistry

**Figure 2:**
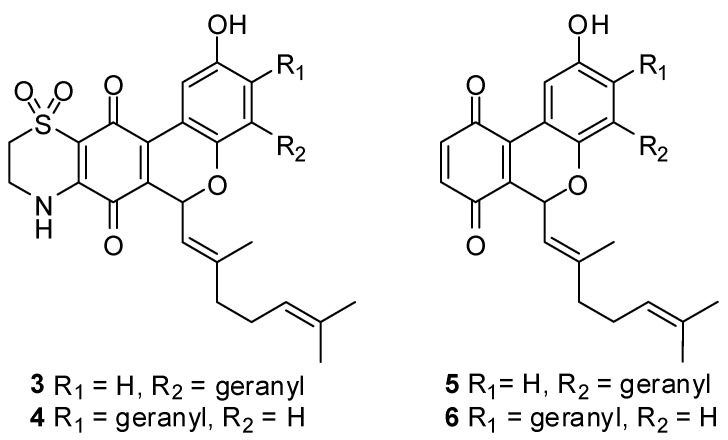
Structures of thiazine regioisomers **3** and **4** and precursor quinones **5** and **6**.

The biomimetic syntheses of thiaplidiaquinones A and B and their corresponding thiazine regioisomers **3** and **4** *via* precursor quinones **5** and **6** (Figure 2) have been reported elsewhere [5].

### 2.2. Biology

#### 2.2.1. Inhibition of ROS Generation

**Figure 3:**
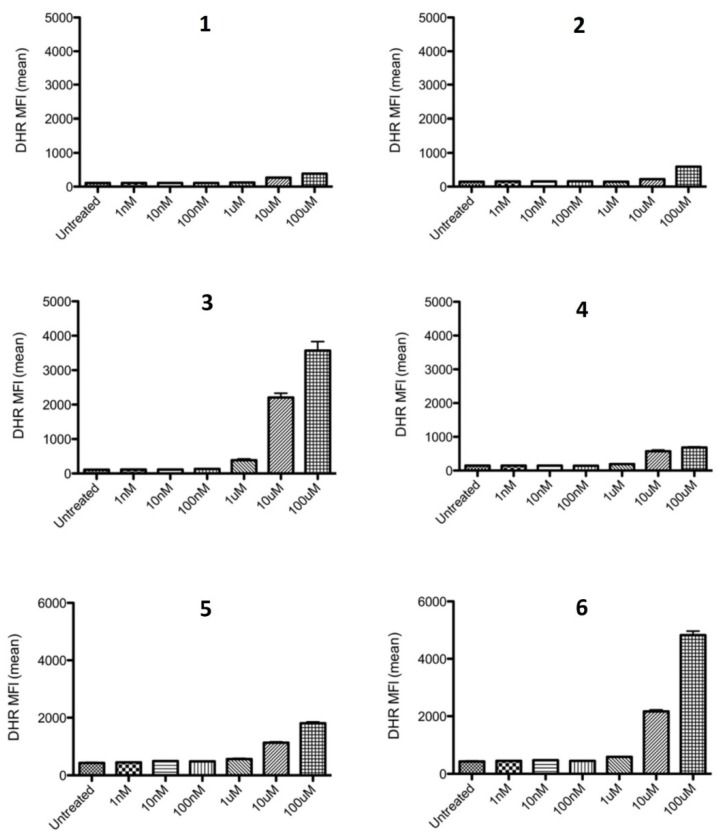
Comparison of DHR fluorescence by Jurkat cells treated with **1**–**6**.

It was originally reported that thiaplidiaquinones A and B displayed a strong accumulation of intracellular ROS in Jurkat cells, 97% of cells for **1** and 93% for **2**, relative to untreated cells [4]. In the present study, the presence of ROS in Jurkat cells was determined using dihydrorhodamine 123 (DHR123), a cell permeable probe that becomes fluorescent when oxidized to rhodamine 123 in the presence of ROS [7]. Once the cells were loaded with DHR123, they were then treated with test compounds at a range of concentrations, and the mean fluorescent intensity determined by flow cytometry. In Figure 3 it is shown that even at a top test concentration of 100 μM, natural products **1** and **2** exhibited only modest levels of accumulation of intracellular ROS, particularly in comparison to precursor quinones **5** and **6** and the dioxothiazine regioisomer of thiaplidiaquinone A **3**. Of the four dioxothiazine-containing compounds (**1**–**4**), **3** was clearly the most potent generator of intracellular ROS in this assay.

#### 2.2.2. Apoptosis *vs.* Necrosis in Jurkat Cells

It was reported previously that natural products **1** and **2** exhibited similar levels of cytotoxicity towards Jurkat cells with IC_50_ ~ 3 μM using a standard cell viability assay [4]. In addition, by using propidium iodide (PI) as a probe, from the observation of treated cells having a significant loss of nuclear DNA in combination with ROS production it was concluded that both **1** and **2** were inducing apoptosis. However, as this approach looked at the average cell population it does not provide the opportunity to clearly differentiate between apoptosing and necrosing cell subpopulations. In our hands, natural products **1** and **2**, and regioisomers **3** and **4**, exhibited significantly less potent cytotoxicity towards Jurkat cells, with toxicity only apparent at concentrations close to 100 μM. For our assessment of the mechanism of cell death, we used the combination of Annexin V-FITC and PI stains to allow us to evaluate the ability of test compounds **1**–**6** to induce apoptosis or necrosis (or both) in Jurkat cells. After 24 h treatment in the presence of 100 μM of each test compound, cells were analyzed by flow cytometry to identify live (AnV^−^/PI^−^), apoptosing (AnV^+^/PI^−^), necrotic (AnV^−^/PI^+^) and late apoptotic (AnV^+^/PI^+^) cells.

**Figure 4:**
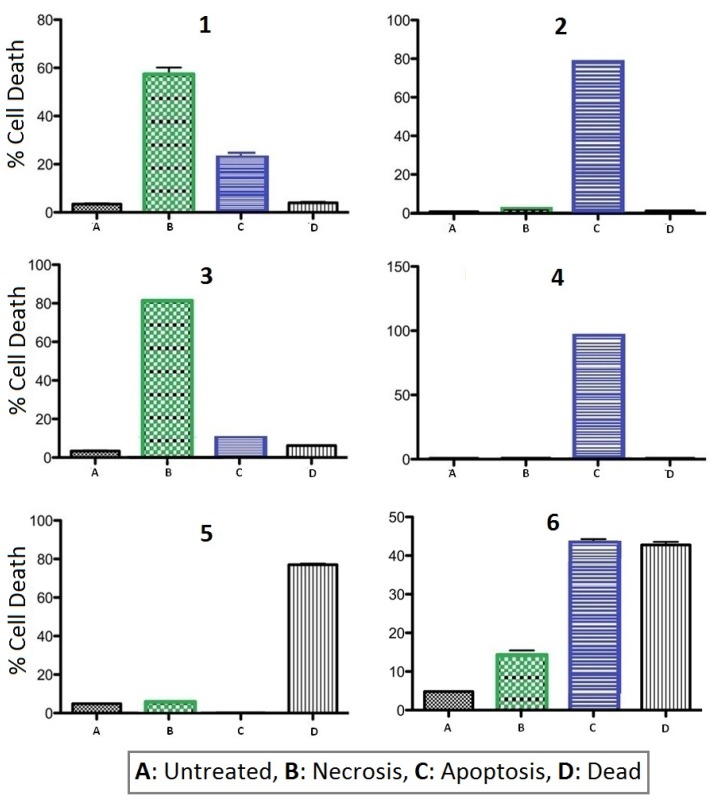
Summary plots of flow cytometry data using Annexin V-FITC and PI to determine mode of cell death for **1**–**6** (necrotic PI^+^/AnV^−^; apoptotic PI^−^/AnV^+^; dead/late apoptosis PI^+^/AnV^−^).

As shown in Figure 4, natural products thiaplidiaquinone A (**1**) and B (**2**) exhibited quite different profiles, with **1** inducing both necrosis (PI^+^/AV^−^) and apoptosis (PI^−^/AV^+^) and **2** causing cell death almost exclusively *via* apoptosis. A similar profile was observed for the dioxothiazine regioisomers **3** and **4**, with an even clearer distinction between cell death by necrosis for **3**, while cell death induced by **4** was dominantly *via* apoptosis. These results identify that placement of the geranyl side chain in ring-D of the molecule specifically dictates the mechanism of cell death (**1**/**3** *vs.* **2**/**4**) with a geranyl chain at the C-3 position resulting in death by apoptosis in comparison to a geranyl chain at the C-4 position which results in necrotic cell death. In direct contrast to the dioxothiazine-containing compounds, the profiles of both precursor quinones **5** and **6** were dominated by late stage apoptosis (PI^+^/AV^+^). From the observations made, it is evident that the presence of the dioxothiazine ring modulates the activity of the compounds (*i.e.*, **1**–**4** *vs.* **5**/**6**) whereas the placement of one of the geranyl side chains in the structure specifically dictates the mechanism of cell death (**1**/**3** *vs.* **2**/**4**). It should be noted that the induction of ROS did not clearly correlate with either apoptotic or necrotic cell death indicating that ROS production appears to be a poor indicator of the route of cell death for the current test compounds. This is not unexpected as necrosis is also linked to ROS production [8,9], thereby confounding the use of ROS as an indicator of the route of cell death.

#### 2.2.3. *In Vitro* Cytotoxicity Screening at the NCI

With the discovery that **1**/**3** and **2**/**4** exhibit contrasting mechanisms of cell death towards Jurkat cells, the set of **1**–**6** were further screened for *in vitro* antitumor activity at the National Cancer Institute [10]. In one dose (10 μM) testing, dioxothiazine regioisomers **3** and **4** exhibited more potent panel average cell kill than the natural products **1** and **2**, with precursor quinones **5** and **6** being found to be the least active (see Supplementary Materials). Selectivity towards melanoma cell lines, in particular MDA-MB-435 (all compounds) and MALME-3M (**3** and **4**), was observed. More comprehensive 5-dose testing of **1**–**4** identified **3**, the regioisomer of thiaplidiaquinone A, to be the more active compound, showing good activity towards MDA-MB-435 (LC_50_ 0.66 μM, TGI 0.18 μM, GI_50_ 0.052 μM) and MALME-3M (LC_50_ 3.4 μM, TGI 0.60 μM, GI_50_ 0.10 μM) cell lines (see Supplementary Materials). This compound has been selected for further *in vivo* evaluation. Our findings that non-natural dioxothiazine regioisomers can exhibit more potent cytotoxicity to tumour cells than the corresponding natural product is in agreement with results observed by Aiello *et al*., as part of their SAR studies of the dioxothiazine-ring containing ascidian natural product aplidinone A [11]. Their study investigated the effect of variation in lipophilic sidechain structure and dioxothiazine ring regiochemistry on a range of biological activities. They identified one structurally simplified analogue of the natural product containing the non-natural dioxothiazine ring regiochemistry that exhibited enhanced cytotoxicity towards a number of tumour cell lines as well as enhanced inhibition of TNFα-induced Nf-κB activation.

#### 2.2.4. Antimalarial and Anti-Farnesyltransferase Activities

Compounds **1**–**6** were evaluated for activity against *Plasmodium falciparum* (NF54 chloroquine sensitive and FcM29-Cameroon chloroquine-resistant strains) and protozoal and human protein farnesyltransferases. The results are presented in Table 1. We have previously determined that the structurally-related marine meroterpene scabellone B exhibited moderate antimalarial activity (IC_50_ 4.8 μM, *Pf* K1 strain) [3]. Evaluation of **1**–**6** against *P. falciparum* identified precursor quinones **5** and **6** to be the most active, followed by regioisomers **3** and **4**, while natural products **1** and **2** were deemed inactive. We have previously determined that the structurally-related marine meroterpene scabellone B exhibited moderate antimalarial activity (IC_50_ 4.8 μM, *Pf* K1 strain). [3] Taken together, these results show that compounds containing only the tricyclic pyranoquinone core structure (*i.e.*, rings B-C-D) such as in compounds **5**, **6** and scabellone B are more active antimalarial agents than those compounds that also contain an additional dioxothiazine ring. The results also suggest that amongst the dioxothiazine-containing compounds **1**–**4**, anti-*Pf* activity is sensitive to the particular regiochemistry of the dioxothiazine ring. With regard to the protein farnesyltransferase (FTase) bioassays, a high degree of correlation was observed between results for FTases of both protozoal and human origin for most of the compounds. In the cases of compounds **2** and **6**, a slightly higher degree of selectivity for human FTase was observed. Of note was the sub-micromolar inhibition of both FTases by the natural product thiaplidiaquinone A (**1**) and regioisomeric analogues **3** and **4**, with the latter exhibiting particularly potent activities (IC_50_ 0.098 and 0.054 μM).

**Table 1:** *In vitro* antimalarial and anti-farnesyltransferase activity of compounds **1**–**6**.

Compound	*P. falc.* ^a^	FTase (*T. b.*) ^b^	FTase (H) ^c^
**1**	>17 ^d^	0.74 ± 0.20	0.78 ± 0.17
**2**	>17 ^d^	3.04 ± 0.30	1.22± 0.068
**3**	4.56 ± 0.76 ^d^	0.22 ± 0.034	0.14 ± 0.0017
**4**	4.39 ± 0.77 ^d^	0.098 ± 0.008	0.054 ± 0.005
**5**	2.2 ^e^	3.90 ± 0.60	3.70 ± 0.60
**6**	2.3 ^e^	6.16 ± 1.40	1.64 ± 0.30
Chloroquine ^f^	0.45 ^d^, 0.0063 ^e^		
FTI 276 ^f^		0.010 ± 0.002	0.015 ± 0.004

IC_50_ values (μM) are reported as the average of three assays with an associated deviation, except for *Pf* data for **5** and **6** which is reported as the average of two independent assays; ^a^ *Plasmodium falciparum*; **^b^** *Trypanosoma brucei* farnesyltransferase; **^c^** Human farnesyltransferase; ^d^ *Plasmodium falciparum*, FcM29-Cameroon strain (chloroquine-resistant); ^e^ *Plasmodium falciparum*, NF54 strain (chloroquine sensitive), IEF stage; ^f^ Chloroquine and FTI 276 were used as positive controls.

## 3. Experimental Section

### 3.1. Chemistry

The syntheses of compounds **1**–**6** have been reported previously [5].

### 3.2. Biology

#### 3.2.1. Cell Culture

Jurkat cells were maintained in RPMI 1640 supplemented with 10% FBS, 1% glutamax and 1% Penicillin/streptomycin. Cultures were maintained at 37 °C with 5% CO_2_, and split 2–3 times per week.

#### 3.2.2. Intracellular ROS

Cells were resuspended in HBSS at 1 × 10^6^ cells/mL and 100 μL cells added to individual wells of a 96-well round bottom plate. DHR123 was added to the wells for a final concentration of 500 nM per well and cells incubated for 5 min to allow uptake of DHR123. Compounds were made up in DMSO to 10 mM for a stock solution. A ten-fold dilution series was made for each stock solution in HBSS from 1 mM to 10 nM and 10 μL of each added to triplicate wells, for final concentrations of 100 μM to 1 nM, along with corresponding DMSO controls. The assay was incubated at 37 °C for 30 min, and the cells were washed and resuspended in FACS buffer (PBS with 0.1% BSA and 0.2% sodium azide). Intracellular ROS was measured by flow cytometry analysis of DHR123 uptake.

#### 3.2.3. Jurkat Cell Cytotoxicity Assay

Cells were resuspended in cRPMI at 1 × 10^6^ cells/mL and 100 μL cells added to individual wells of a 96-well round bottom plate. Compounds were made up in DMSO to 10 mM for a stock solution. A ten-fold dilution series was made for each stock solution in cRPMI from 1 mM to 10 nM and 10 μL of each added to triplicate wells, for final concentrations of 100 μM to 1 nM, along with corresponding DMSO controls. The cells plus compounds were then incubated for 24 h at 37 °C. The cells were washed in ice-cold Annexin V binding buffer (200 mL PBS with 10 mM HEPES, 140 mM NaCl and 2.5 mM CaCl_2_). The cell pellets were labelled with 5 μL of Annexin V-FITC (BD Pharmingen) for 15 min at 4 °C. Cells were washed again with ice-cold Annexin V buffer and labelled with 10 μL of 5 μg/mL PI for 7 min at 4 °C. Cells were washed and resuspended in FACS buffer, and Annexin V/PI staining analysed using flow cytometry. Annexin V positive cells were undergoing apoptosis, while PI positive cells were necrotic. Double positive cells were dead, while double negative cells were live cells.

#### 3.2.4. NCI Evaluation

Detailed protocols have been reported elsewhere [12].

#### 3.2.5. Antimalarial and Anti-Farnesyltransferase Evaluation

Detailed protocols have been reported elsewhere [13,14].

## 4. Conclusions

Our biological evaluation of members of a small library of compounds focused on the marine meroterpenoids thiaplidiaquinone A and B has revealed that the non-natural dioxothiazine regioisomers **3** and **4** tend to exhibit more potent biological activities against a number of cellular targets. An unexpected discovery was that the mechanism of Jurkat cell death was dominantly *via* necrosis for thiaplidiaquinone A but *via* apoptosis for the geranyl sidechain positional isomer thiaplidiaquinone B. The finding that the dioxothiazine regioisomers of the natural products exhibited more potent activities in a range of bioassays highlights the crucial role that total synthesis can play in the discovery of new bioactive scaffolds.

## Supplementary Files

Supplementary File 1
