# Supplementary material for: Structure-Activity Relationships of the Bioactive Thiazinoquinone Marine Natural Products Thiaplidiaquinones A and B
Source: Mar Drugs. 2015 Aug 10;13(8):5102–10. doi: 10.3390/md13085102 (PMC4557015; doi:10.3390/md13085102)
Supplement: Supplementary File 1 [file marinedrugs-13-05102-s001.docx]

**Supplementray Information**


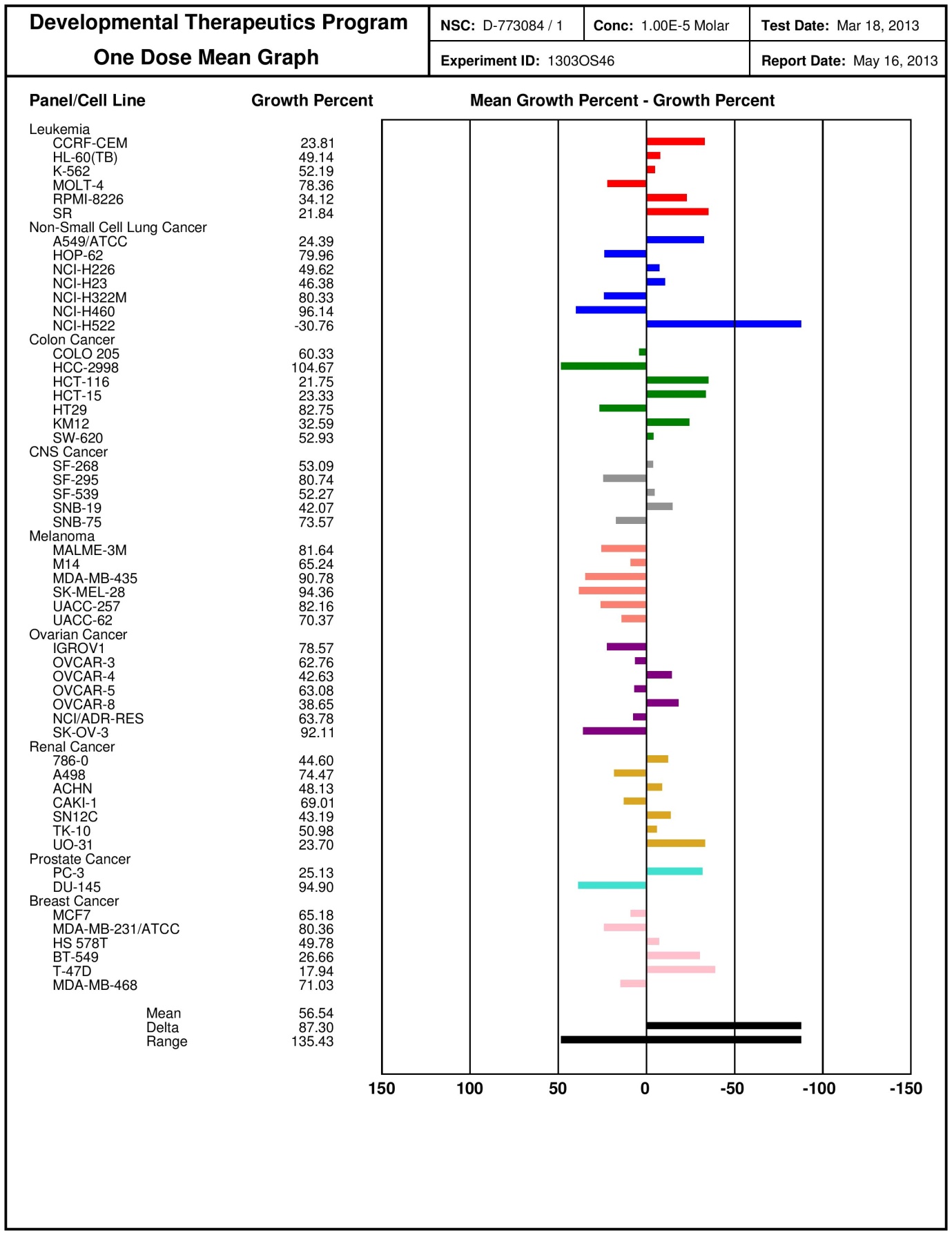


**Figure S1.** Results of NCI-60 cell line assay at one dose against precursor to thiaplidiaquinone A (**5**).


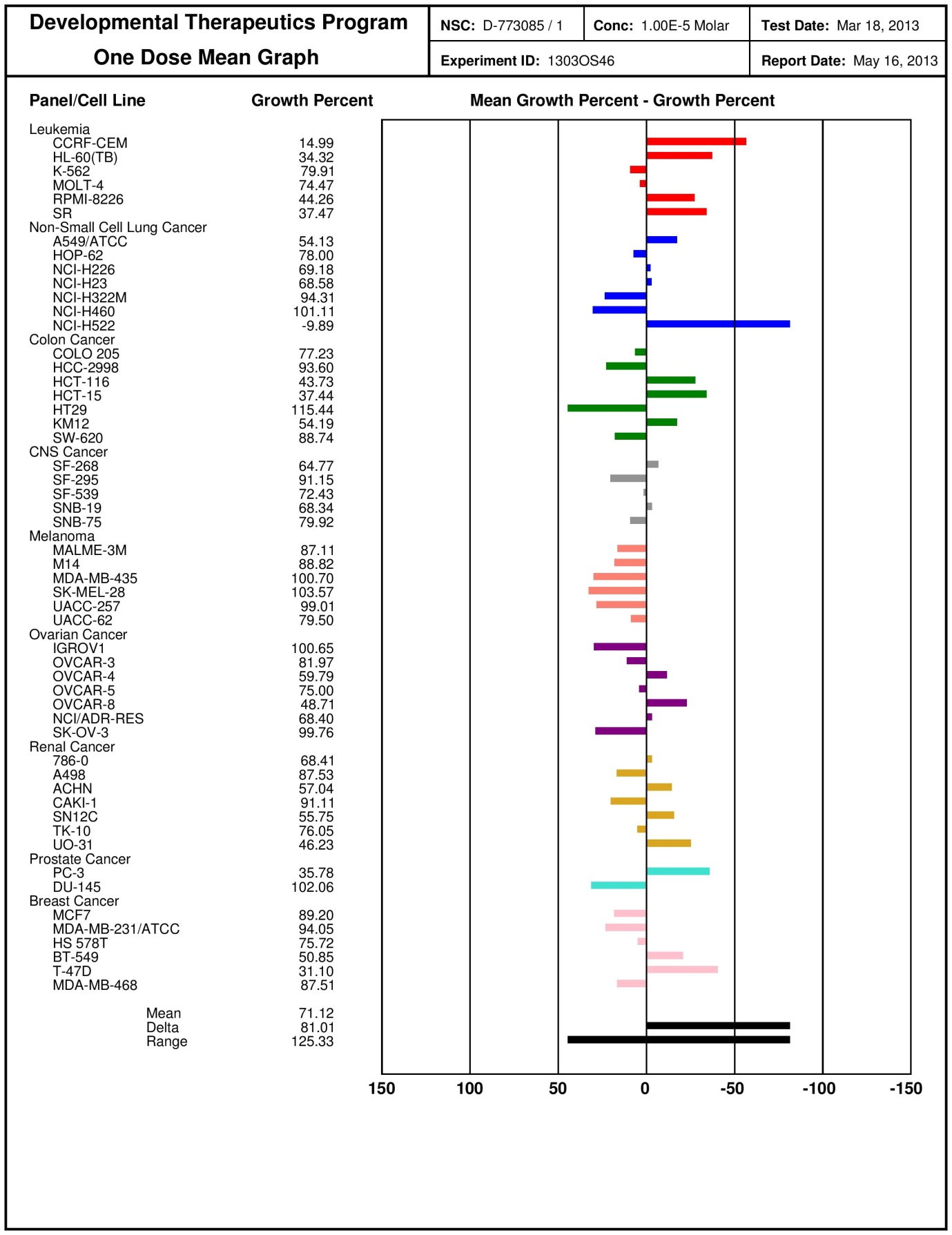


**Figure S2.** Results of NCI-60 cell line assay at one dose against precursor to thiaplidiaquinone B (**6**).


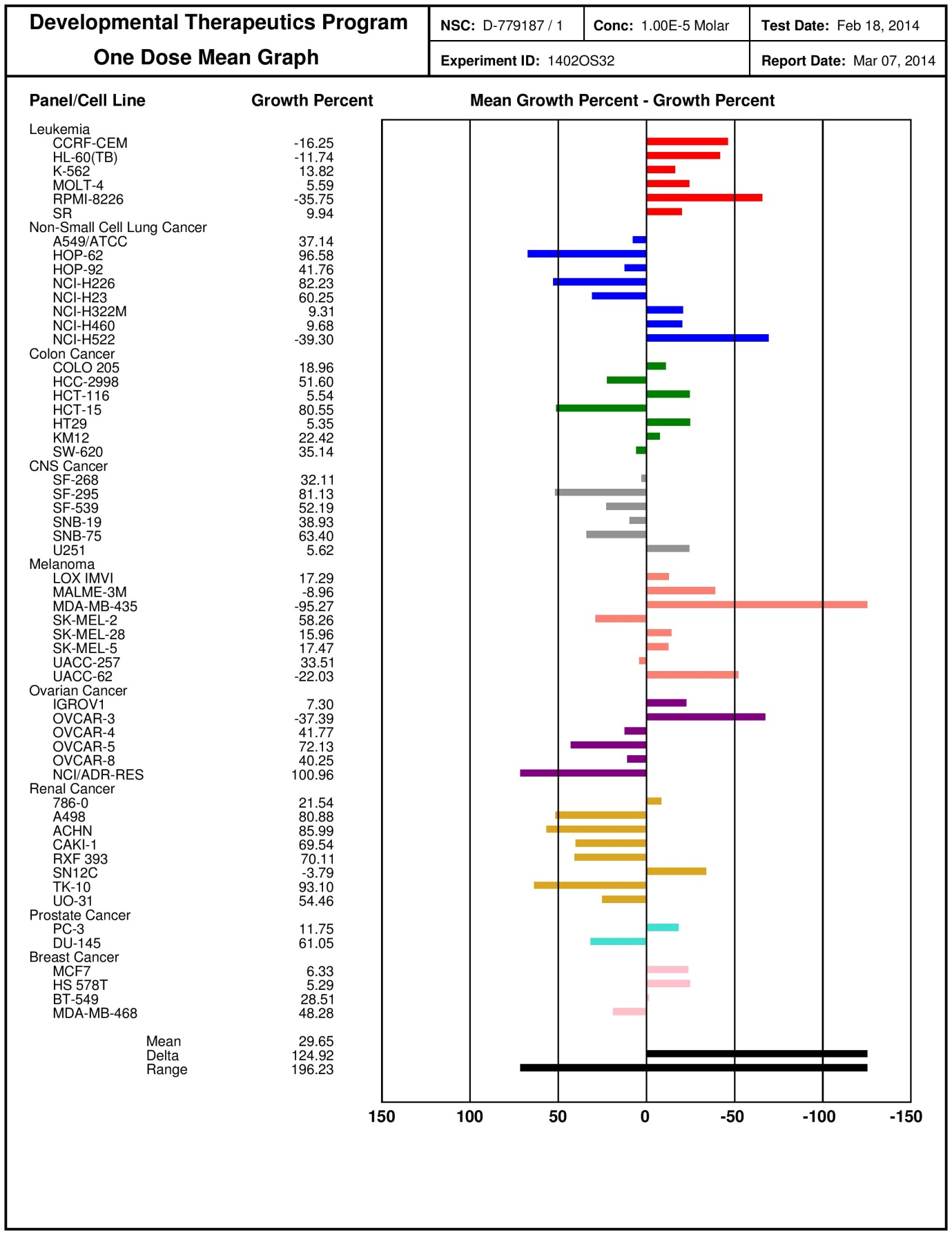


**Figure S3.** Results of NCI-60 cell line assay at one dose against thiaplidiaquinone A (**1**).


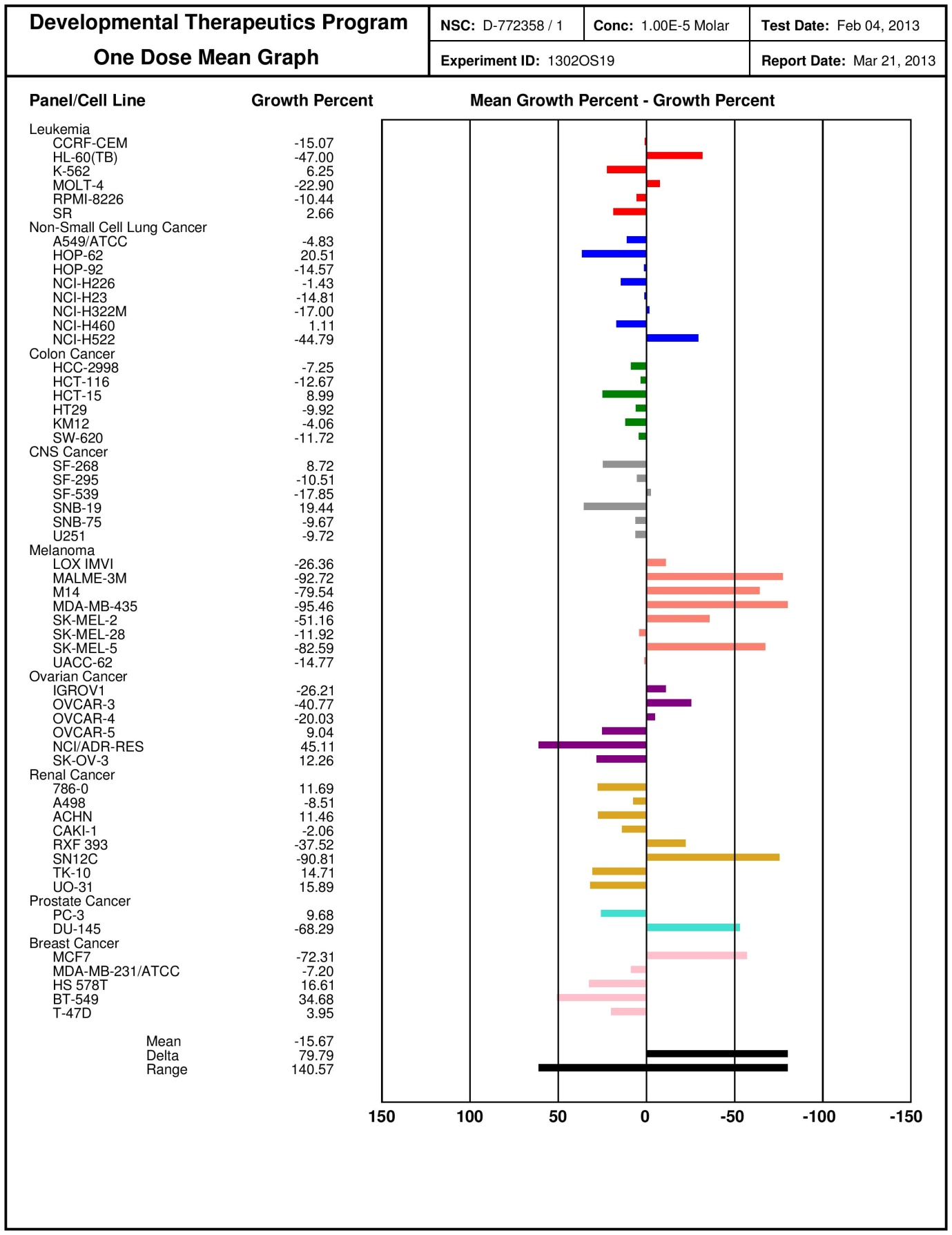


**Figure S4.** Results of NCI-60 cell line assay at one dose against regioisomer of thiaplidiaquinone A (**3**).


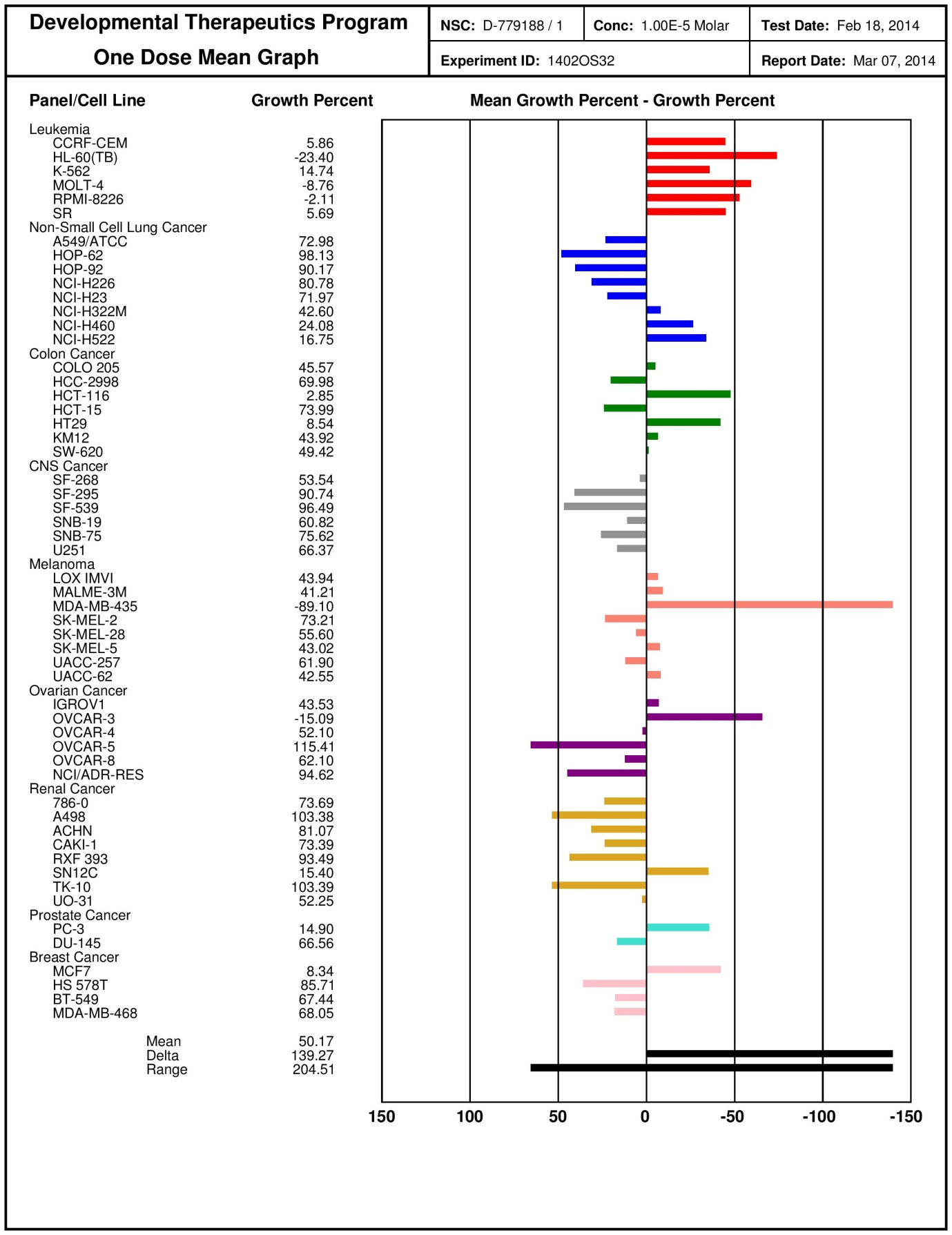


**Figure S5.** Results of NCI-60 cell line assay at one dose against thiaplidiaquinone B (**2**).


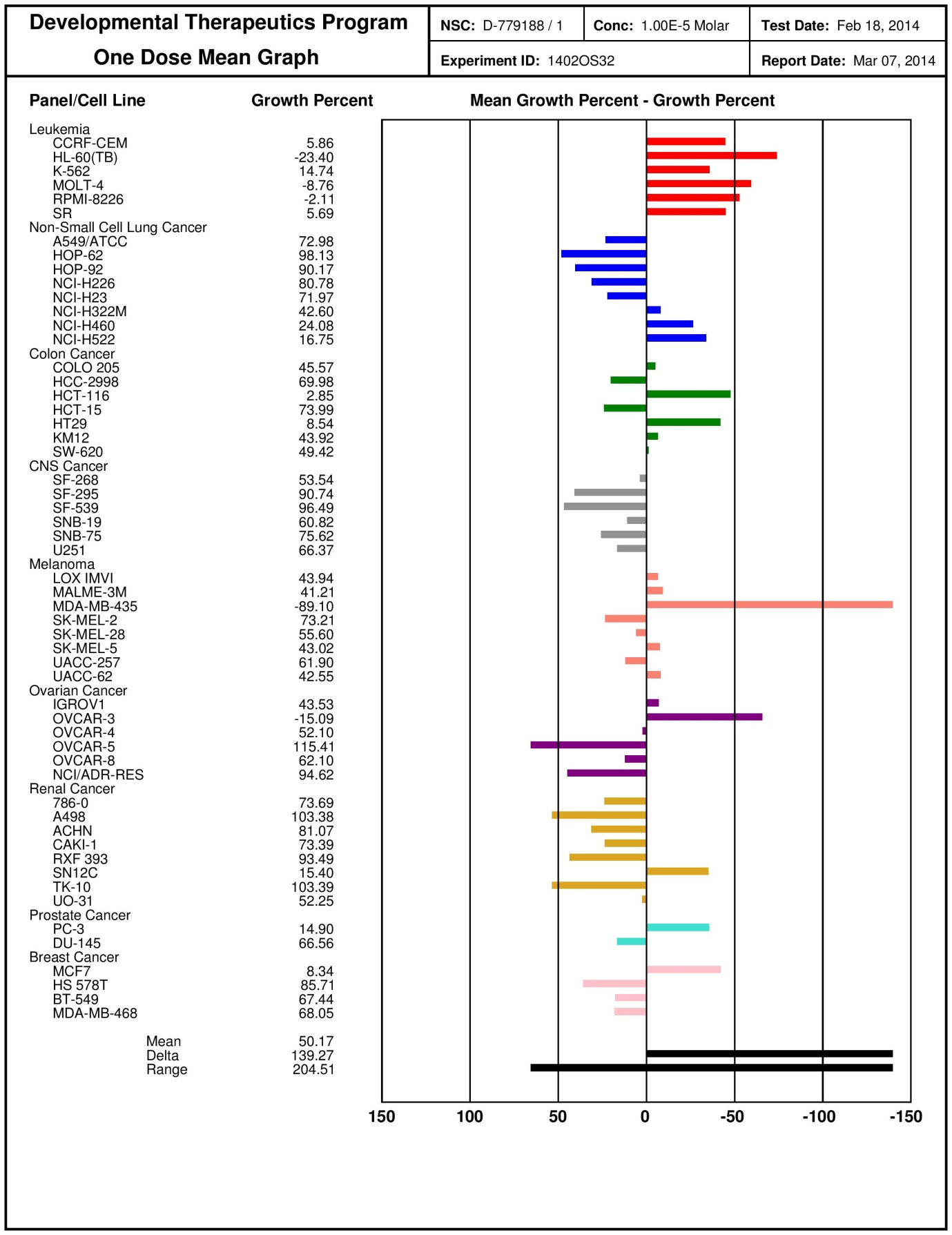


**Figure S6.** Results of NCI-60 cell line assay at one dose against regioisomer of thiaplidiaquinone B (**4**).


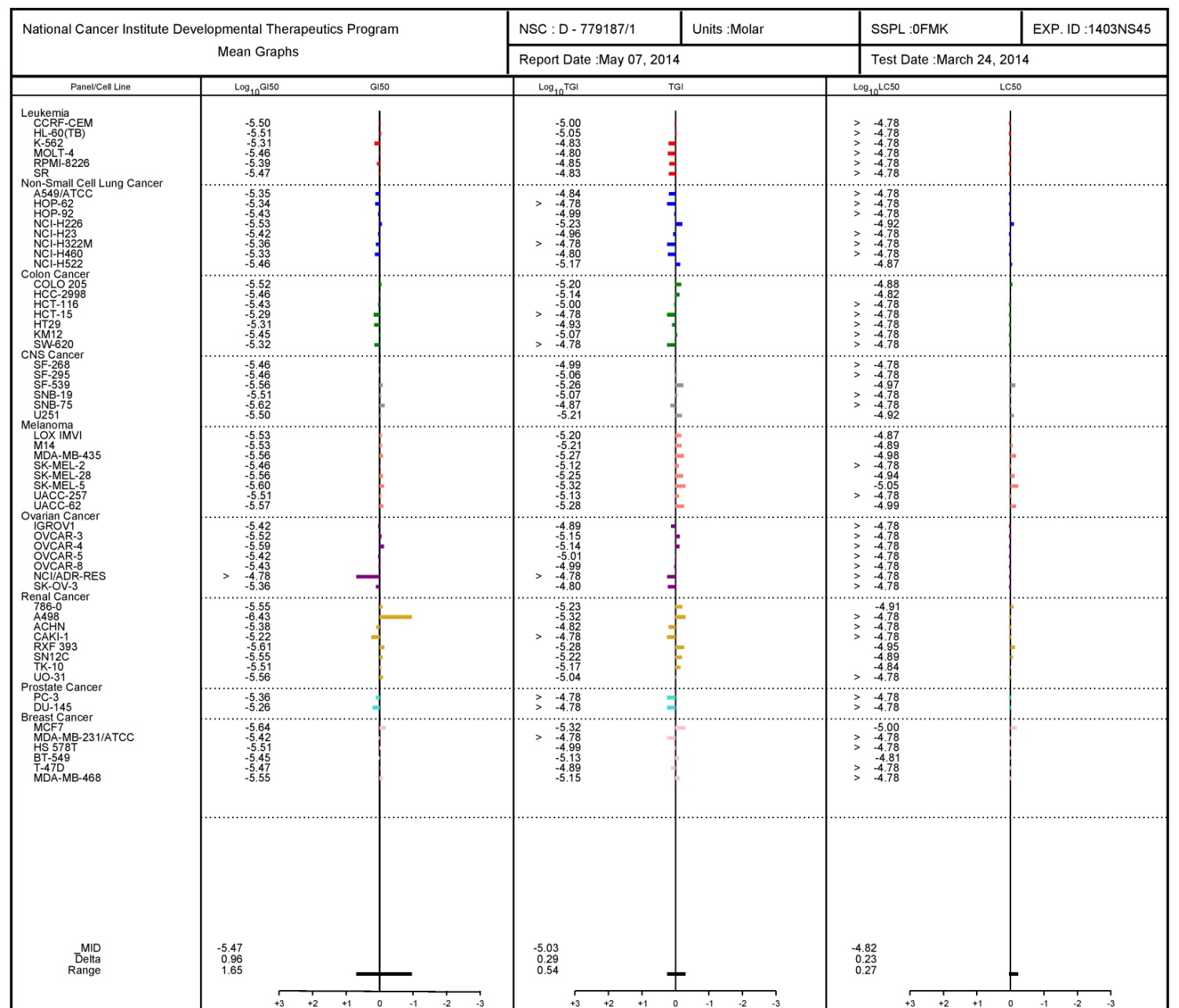


**Figure S7.** Results of NCI-60 cell line assay at five dose testing against thiaplidiaquinone A (**1**).


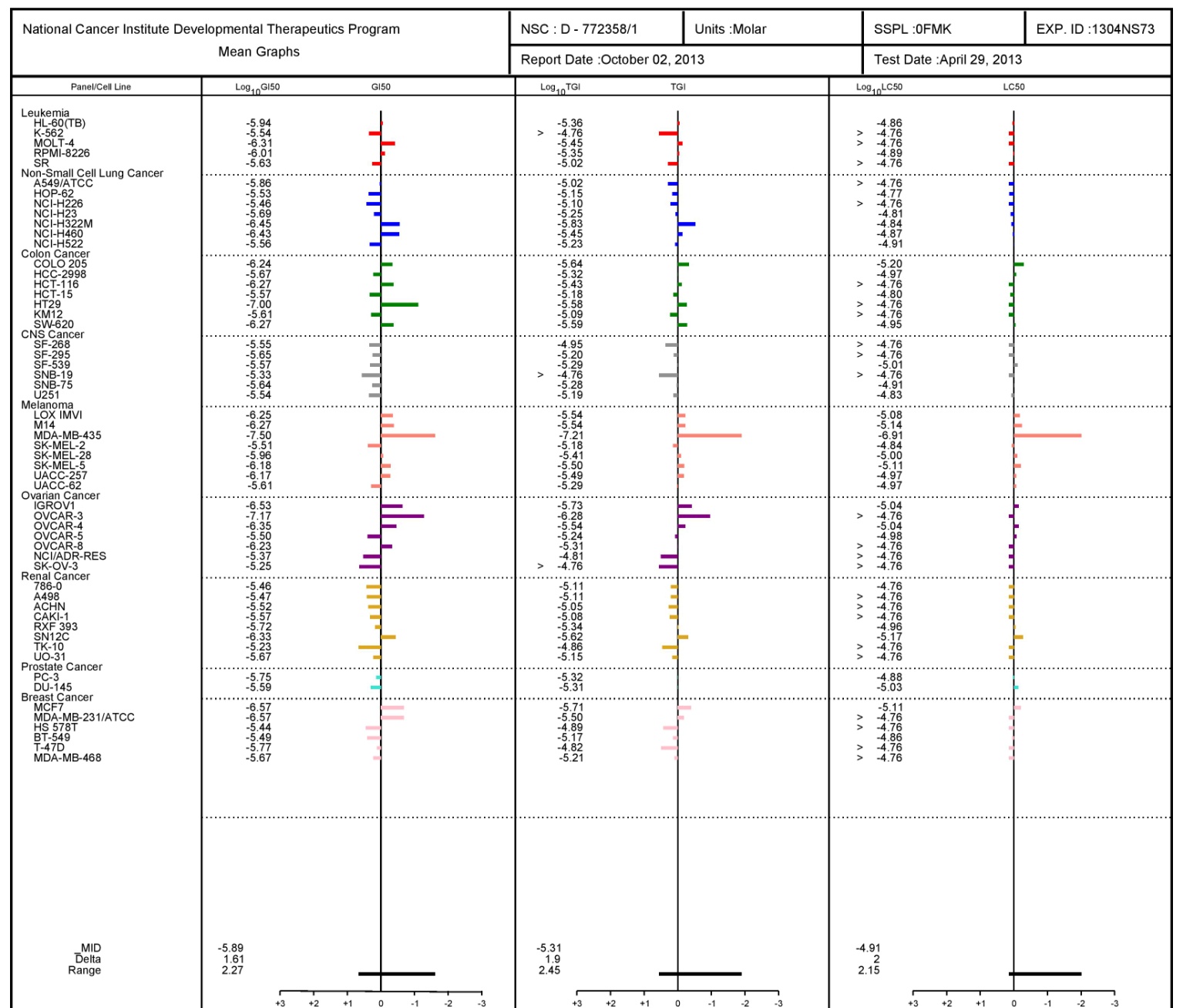


**Figure S8.** Results of NCI-60 cell line assay at five dose testing against the regioisomer of thiaplidiaquinone A (**3**).


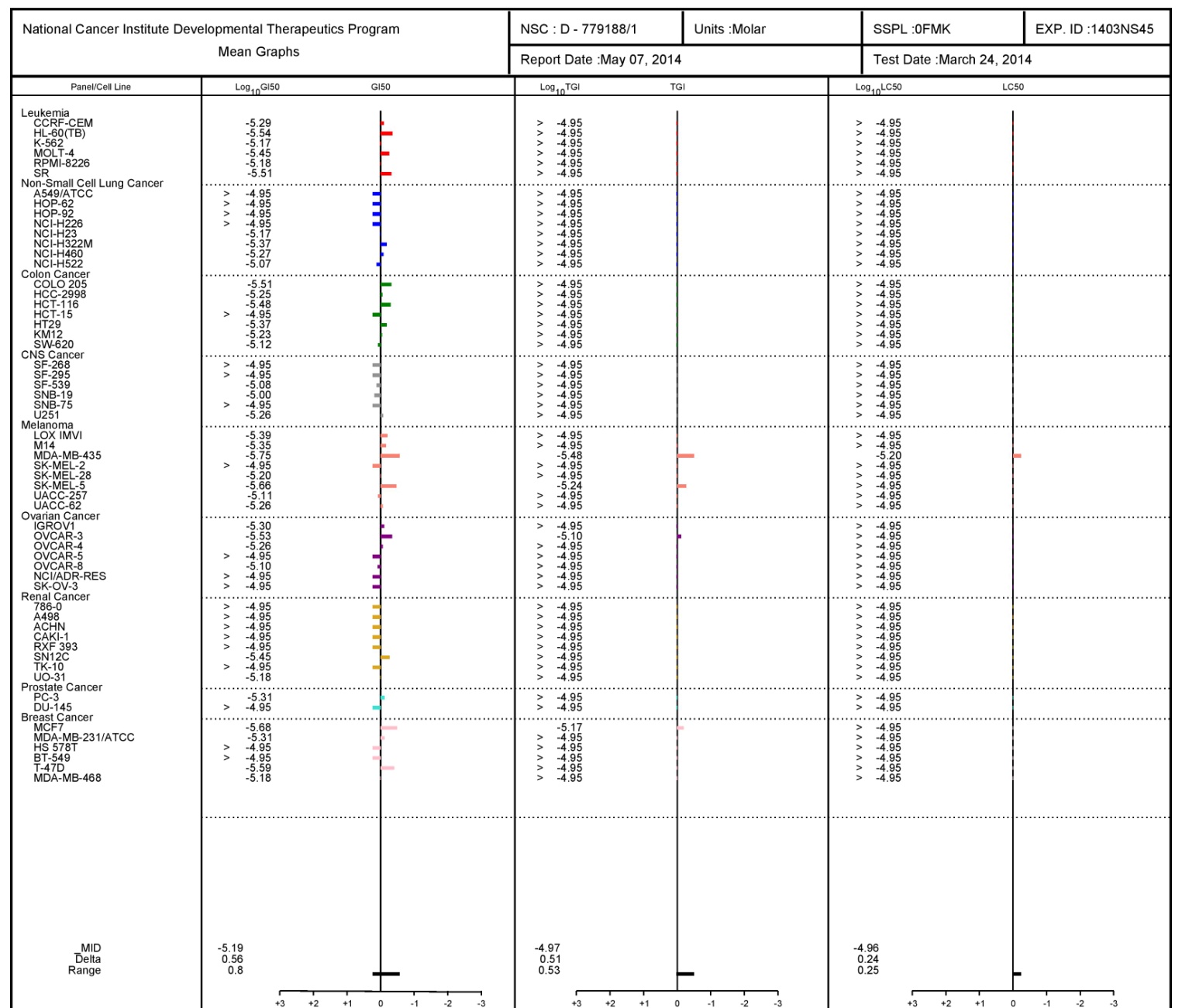


**Figure S9.** Results of NCI-60 cell line assay at five dose testing against thiaplidiaquinone B (**2**).


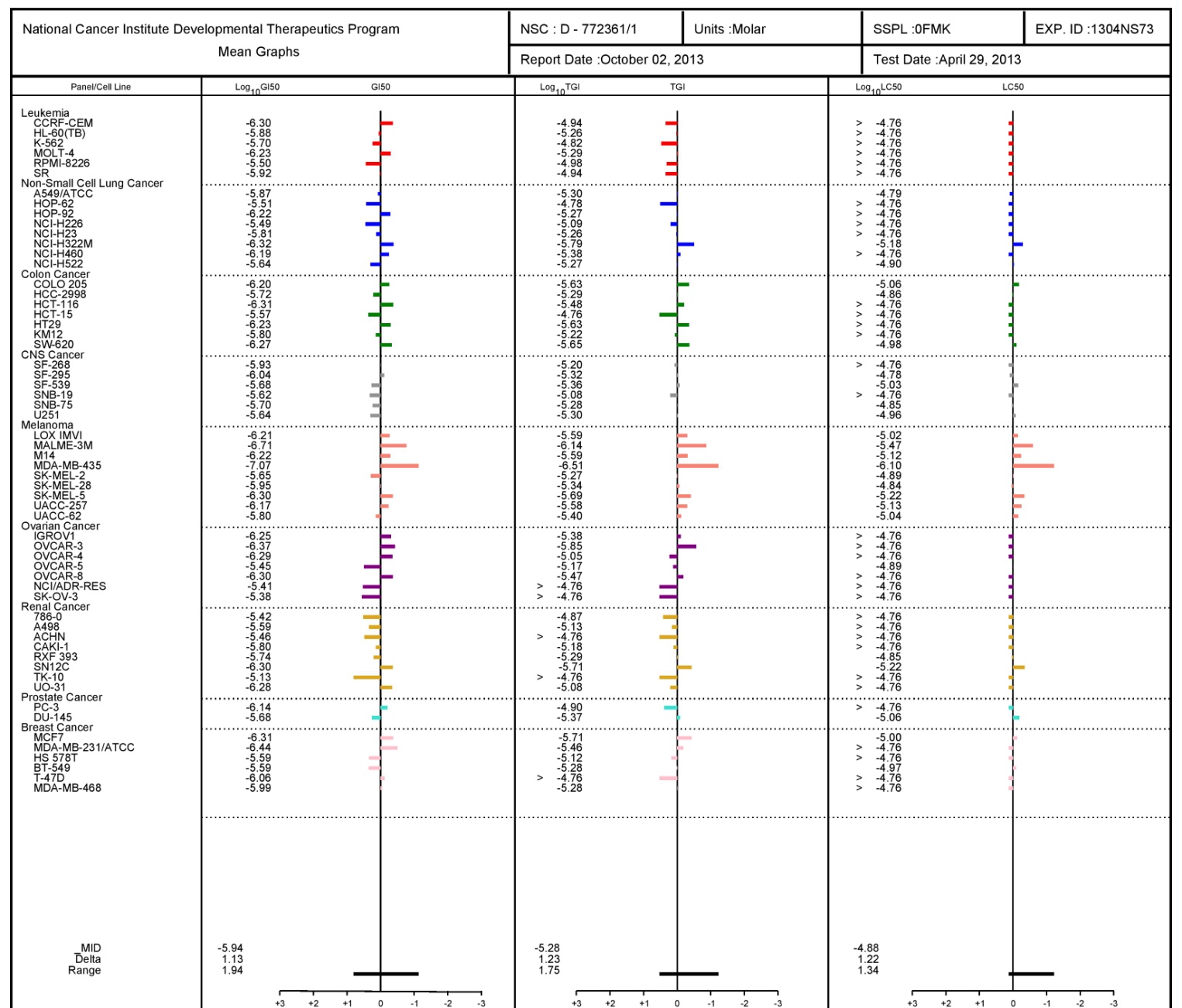


**Figure S10.** Results of NCI-60 cell line assay at five dose testing against the regioisomer of thiaplidiaquinone B (**4**).

© 2015 by the authors; licensee MDPI, Basel, Switzerland. This article is an open access article distributed under the terms and conditions of the Creative Commons Attribution license (http://creativecommons.org/licenses/by/4.0/).
